# Supplementary material for: Multiplexed Imaging Reveals the Spatial Relationship of the Extracellular Acidity-Targeting pHLIP with Necrosis, Hypoxia, and the Integrin-Targeting cRGD Peptide
Source: Cells. 2022 Nov 4;11(21):3499. doi: 10.3390/cells11213499 (PMC9656628; doi:10.3390/cells11213499)
Supplement: Supplementary file 1 [file cells-11-03499-s001.zip › cells-1837253-supplementary.pptx]

## Slide 1
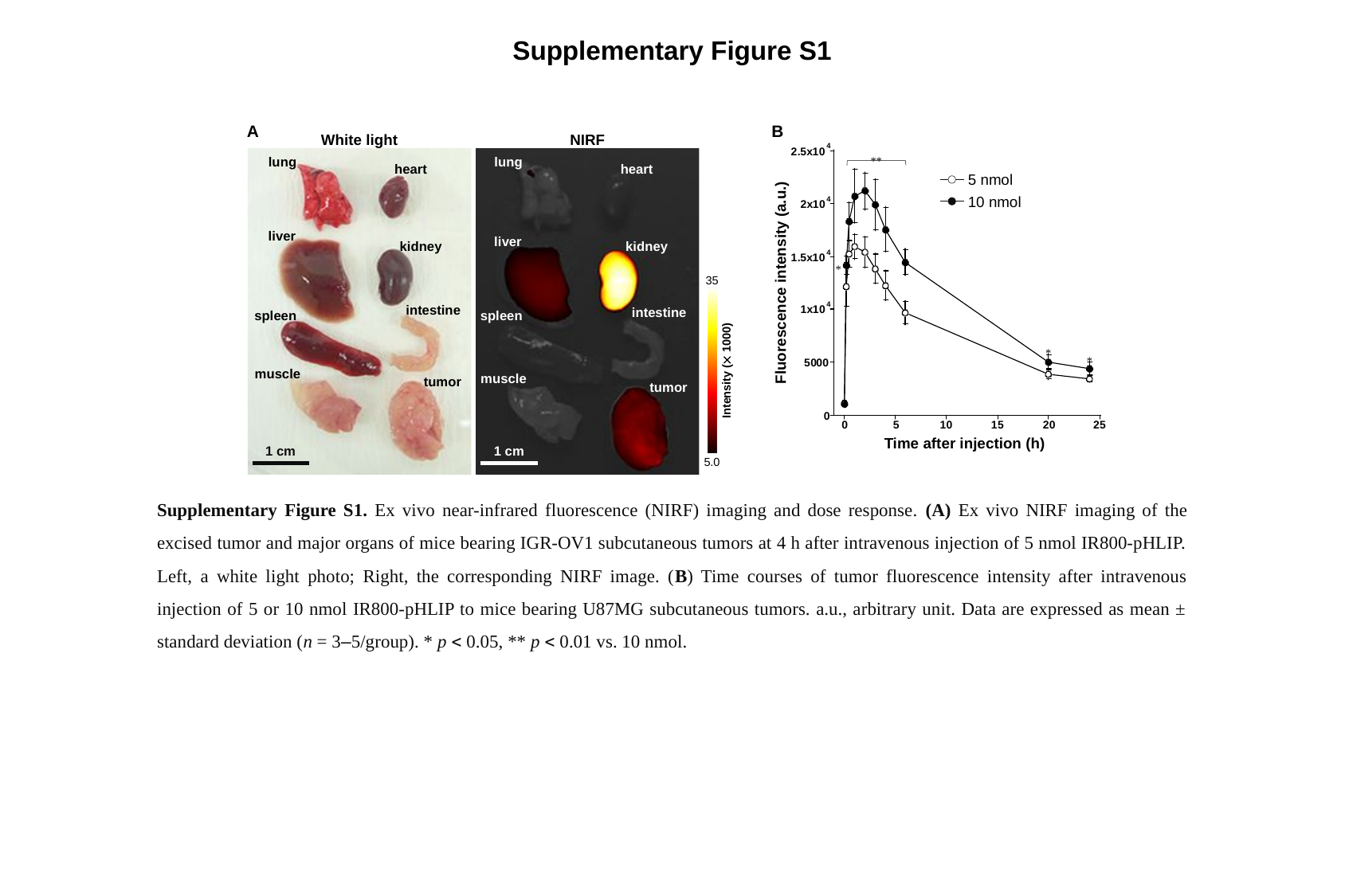

Supplementary Figure S1
A
White light
NIRF
lung
heart
liver
kidney
intestine
spleen
muscle
tumor
1 cm
lung
heart
liver
kidney
intestine
spleen
muscle
tumor
1 cm
35
Intensity (× 1000)
5.0
B
**
5 nmol
10 nmol
*
Fluorescence intensity (a.u.)
*
*
Time after injection (h)
Supplementary Figure S1. Ex vivo near-infrared fluorescence (NIRF) imaging and dose response. (A) Ex vivo NIRF imaging of the excised tumor and major organs of mice bearing IGR-OV1 subcutaneous tumors at 4 h after intravenous injection of 5 nmol IR800-pHLIP. Left, a white light photo; Right, the corresponding NIRF image. (B) Time courses of tumor fluorescence intensity after intravenous injection of 5 or 10 nmol IR800-pHLIP to mice bearing U87MG subcutaneous tumors. a.u., arbitrary unit. Data are expressed as mean ± standard deviation (n = 3–5/group). * p  0.05, ** p  0.01 vs. 10 nmol.

## Slide 2
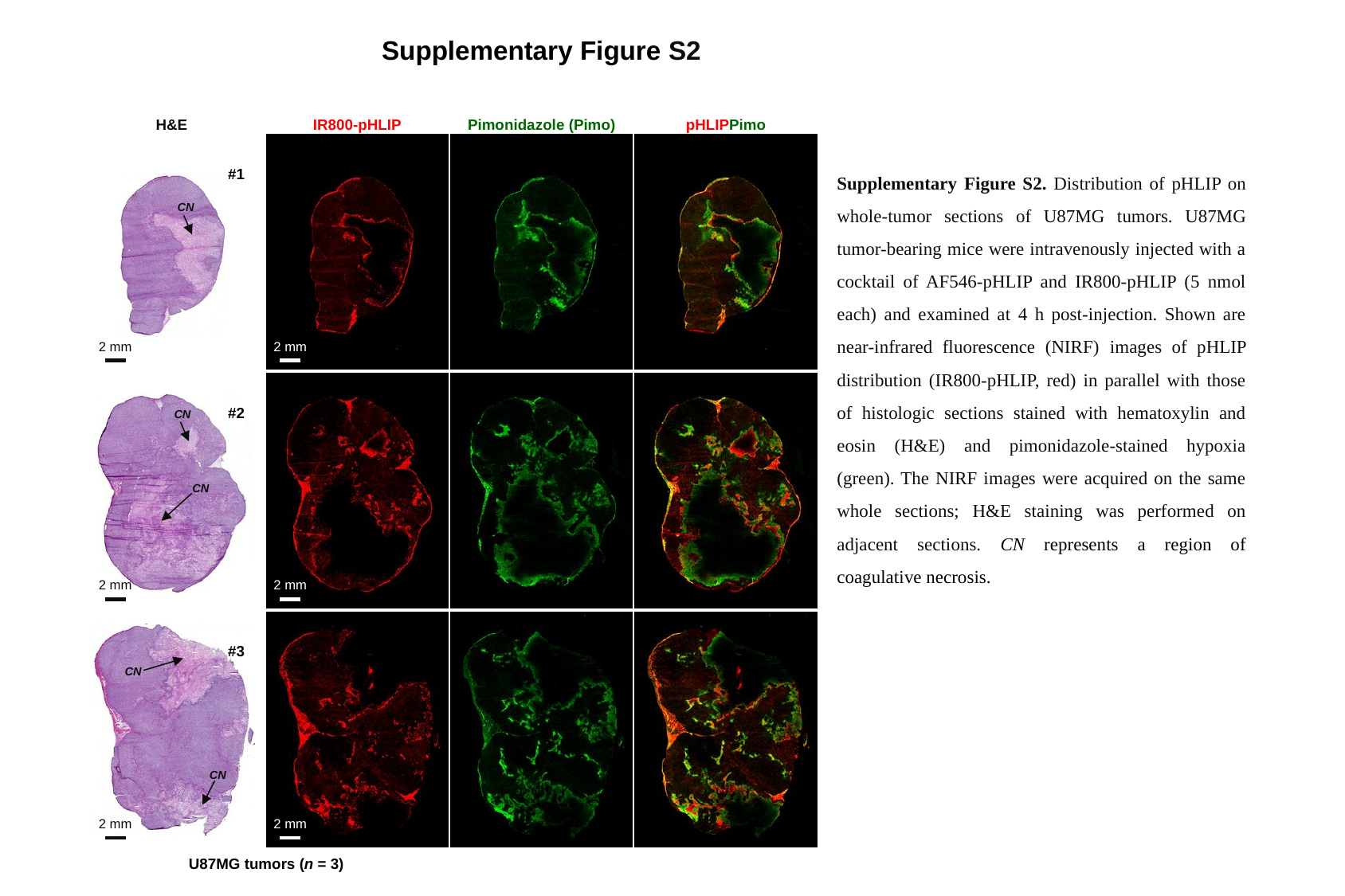

Supplementary Figure S2
H&E
IR800-pHLIP
Pimonidazole (Pimo)
pHLIPPimo
#1
CN
2 mm
2 mm
#2
CN
CN
2 mm
2 mm
#3
CN
CN
2 mm
2 mm
U87MG tumors (n = 3)
Supplementary Figure S2. Distribution of pHLIP on whole-tumor sections of U87MG tumors. U87MG tumor-bearing mice were intravenously injected with a cocktail of AF546-pHLIP and IR800-pHLIP (5 nmol each) and examined at 4 h post-injection. Shown are near-infrared fluorescence (NIRF) images of pHLIP distribution (IR800-pHLIP, red) in parallel with those of histologic sections stained with hematoxylin and eosin (H&E) and pimonidazole-stained hypoxia (green). The NIRF images were acquired on the same whole sections; H&E staining was performed on adjacent sections. CN represents a region of coagulative necrosis.

## Slide 3
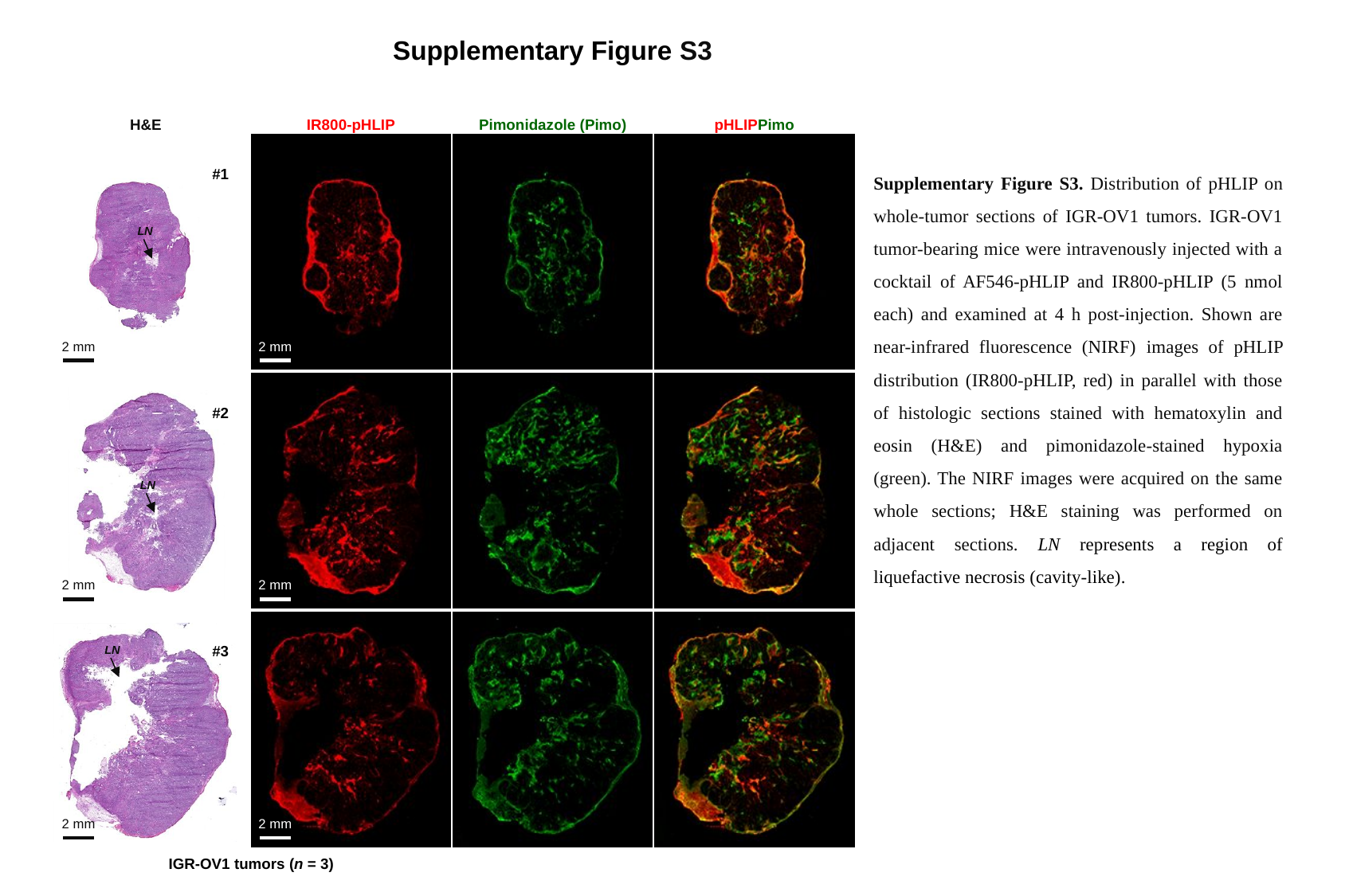

Supplementary Figure S3
H&E
IR800-pHLIP
Pimonidazole (Pimo)
pHLIPPimo
#1
LN
2 mm
2 mm
#2
LN
2 mm
2 mm
#3
LN
2 mm
2 mm
IGR-OV1 tumors (n = 3)
Supplementary Figure S3. Distribution of pHLIP on whole-tumor sections of IGR-OV1 tumors. IGR-OV1 tumor-bearing mice were intravenously injected with a cocktail of AF546-pHLIP and IR800-pHLIP (5 nmol each) and examined at 4 h post-injection. Shown are near-infrared fluorescence (NIRF) images of pHLIP distribution (IR800-pHLIP, red) in parallel with those of histologic sections stained with hematoxylin and eosin (H&E) and pimonidazole-stained hypoxia (green). The NIRF images were acquired on the same whole sections; H&E staining was performed on adjacent sections. LN represents a region of liquefactive necrosis (cavity-like).

## Slide 4
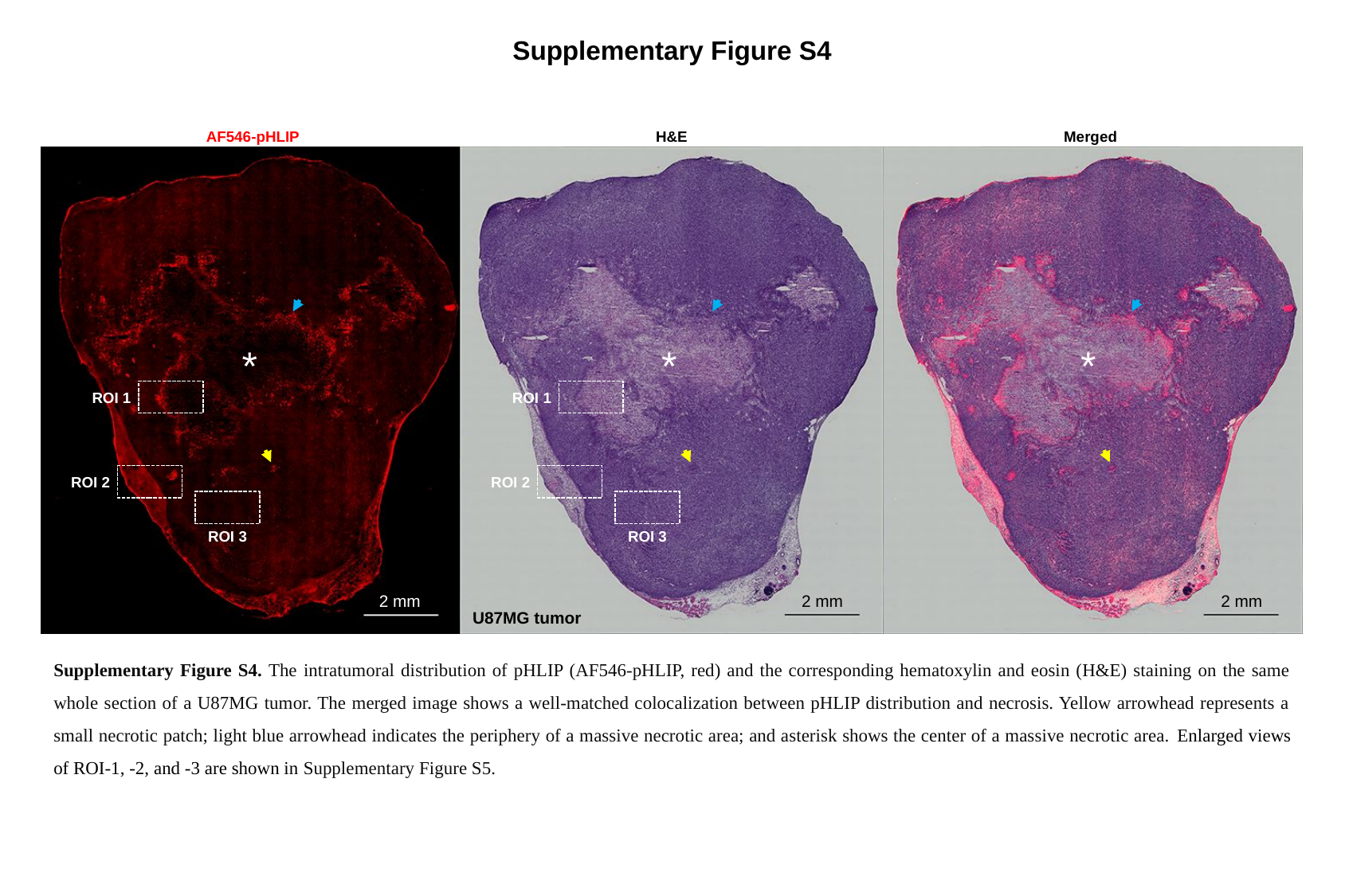

Supplementary Figure S4
AF546-pHLIP
*
ROI 1
ROI 2
ROI 3
2 mm
H&E
*
ROI 1
ROI 2
ROI 3
2 mm
U87MG tumor
Merged
*
2 mm
Supplementary Figure S4. The intratumoral distribution of pHLIP (AF546-pHLIP, red) and the corresponding hematoxylin and eosin (H&E) staining on the same whole section of a U87MG tumor. The merged image shows a well-matched colocalization between pHLIP distribution and necrosis. Yellow arrowhead represents a small necrotic patch; light blue arrowhead indicates the periphery of a massive necrotic area; and asterisk shows the center of a massive necrotic area. Enlarged views of ROI-1, -2, and -3 are shown in Supplementary Figure S5.

## Slide 5
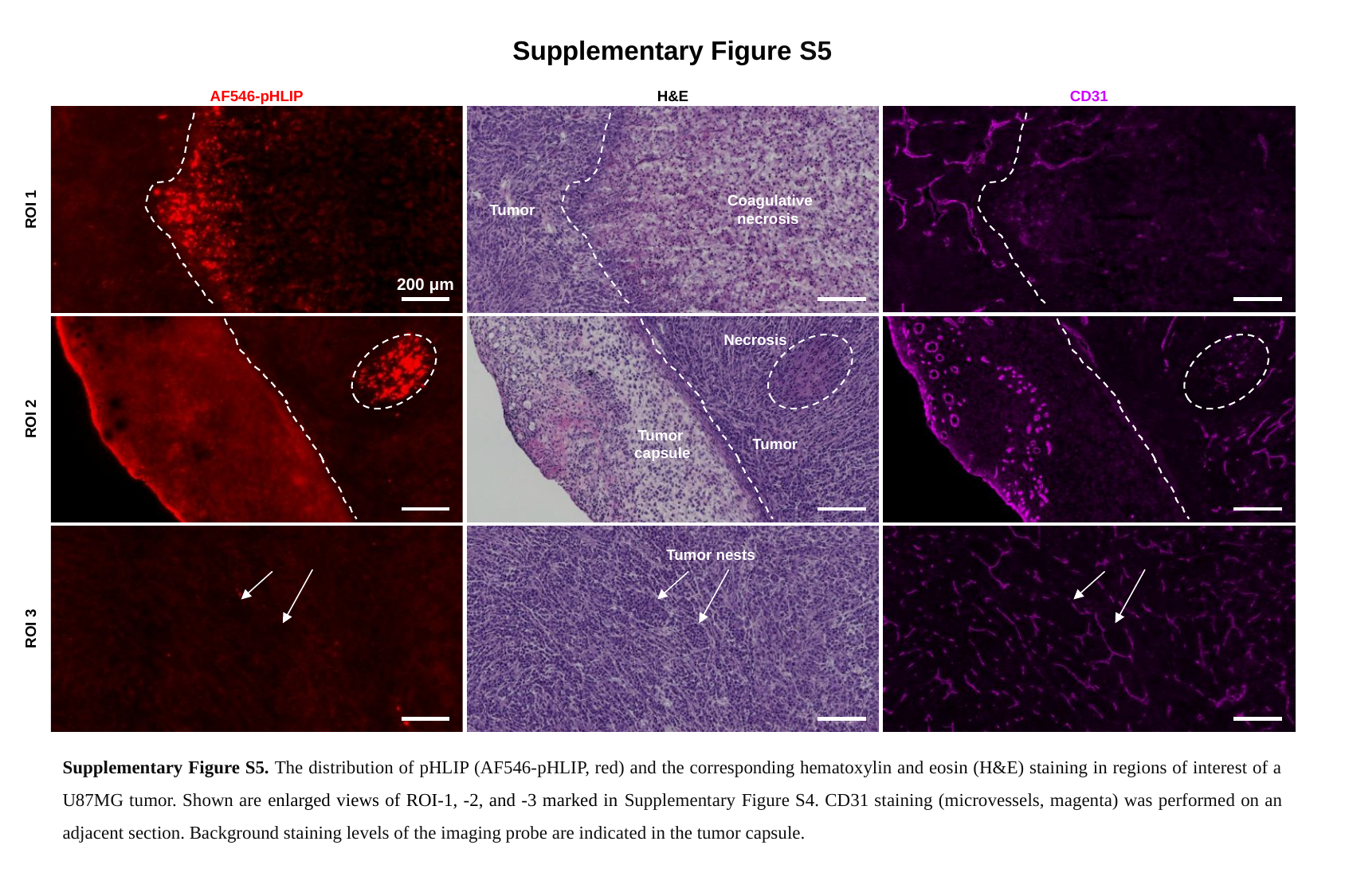

Supplementary Figure S5
AF546-pHLIP
H&E
CD31
Coagulative
necrosis
Tumor
ROI 1
200 μm
Necrosis
ROI 2
Tumor
capsule
Tumor
Tumor nests
ROI 3
Supplementary Figure S5. The distribution of pHLIP (AF546-pHLIP, red) and the corresponding hematoxylin and eosin (H&E) staining in regions of interest of a U87MG tumor. Shown are enlarged views of ROI-1, -2, and -3 marked in Supplementary Figure S4. CD31 staining (microvessels, magenta) was performed on an adjacent section. Background staining levels of the imaging probe are indicated in the tumor capsule.

## Slide 6
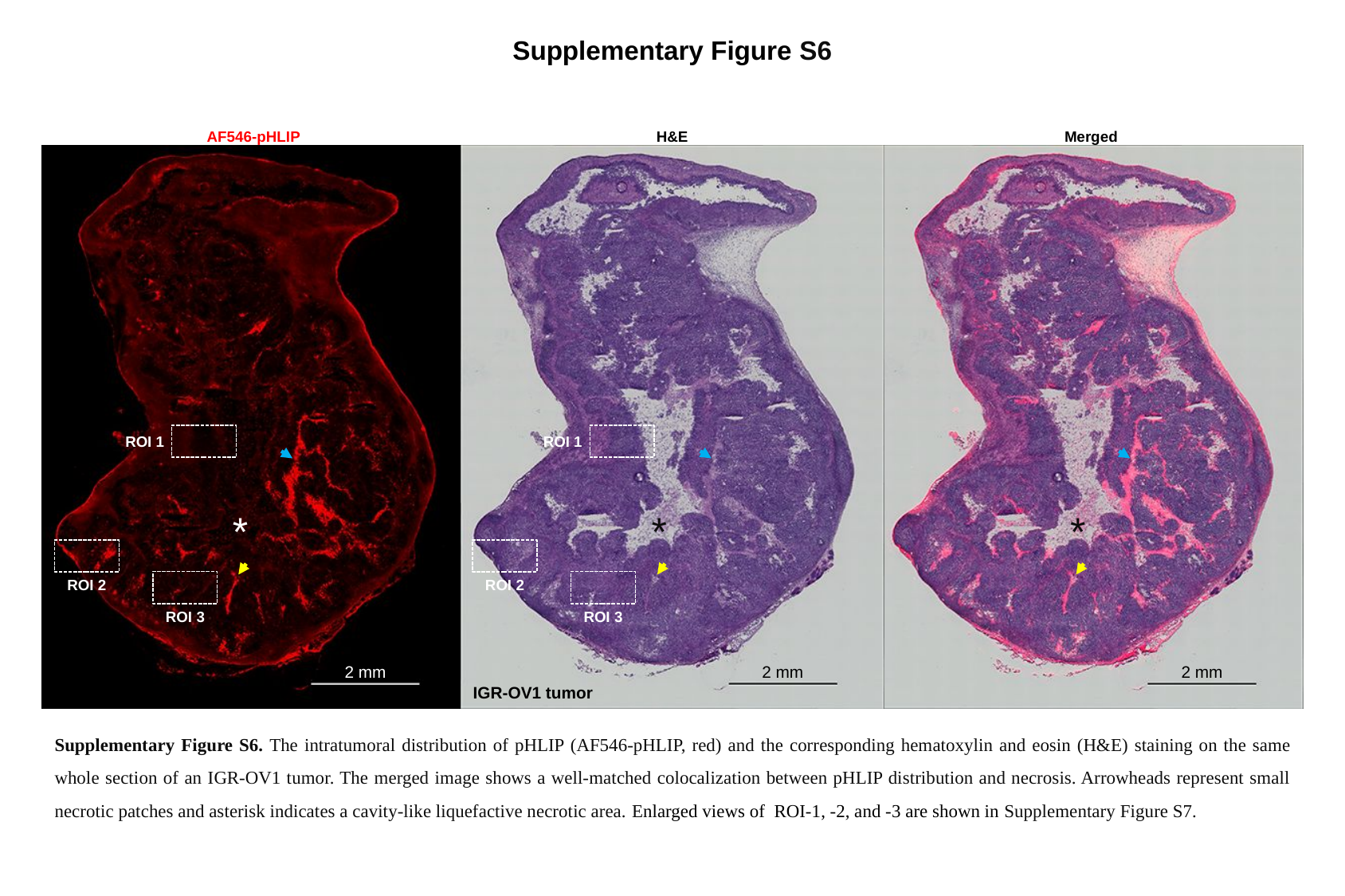

Supplementary Figure S6
AF546-pHLIP
ROI 1
*
ROI 2
ROI 3
2 mm
H&E
ROI 1
*
ROI 2
ROI 3
2 mm
IGR-OV1 tumor
Merged
*
2 mm
Supplementary Figure S6. The intratumoral distribution of pHLIP (AF546-pHLIP, red) and the corresponding hematoxylin and eosin (H&E) staining on the same whole section of an IGR-OV1 tumor. The merged image shows a well-matched colocalization between pHLIP distribution and necrosis. Arrowheads represent small necrotic patches and asterisk indicates a cavity-like liquefactive necrotic area. Enlarged views of ROI-1, -2, and -3 are shown in Supplementary Figure S7.

## Slide 7
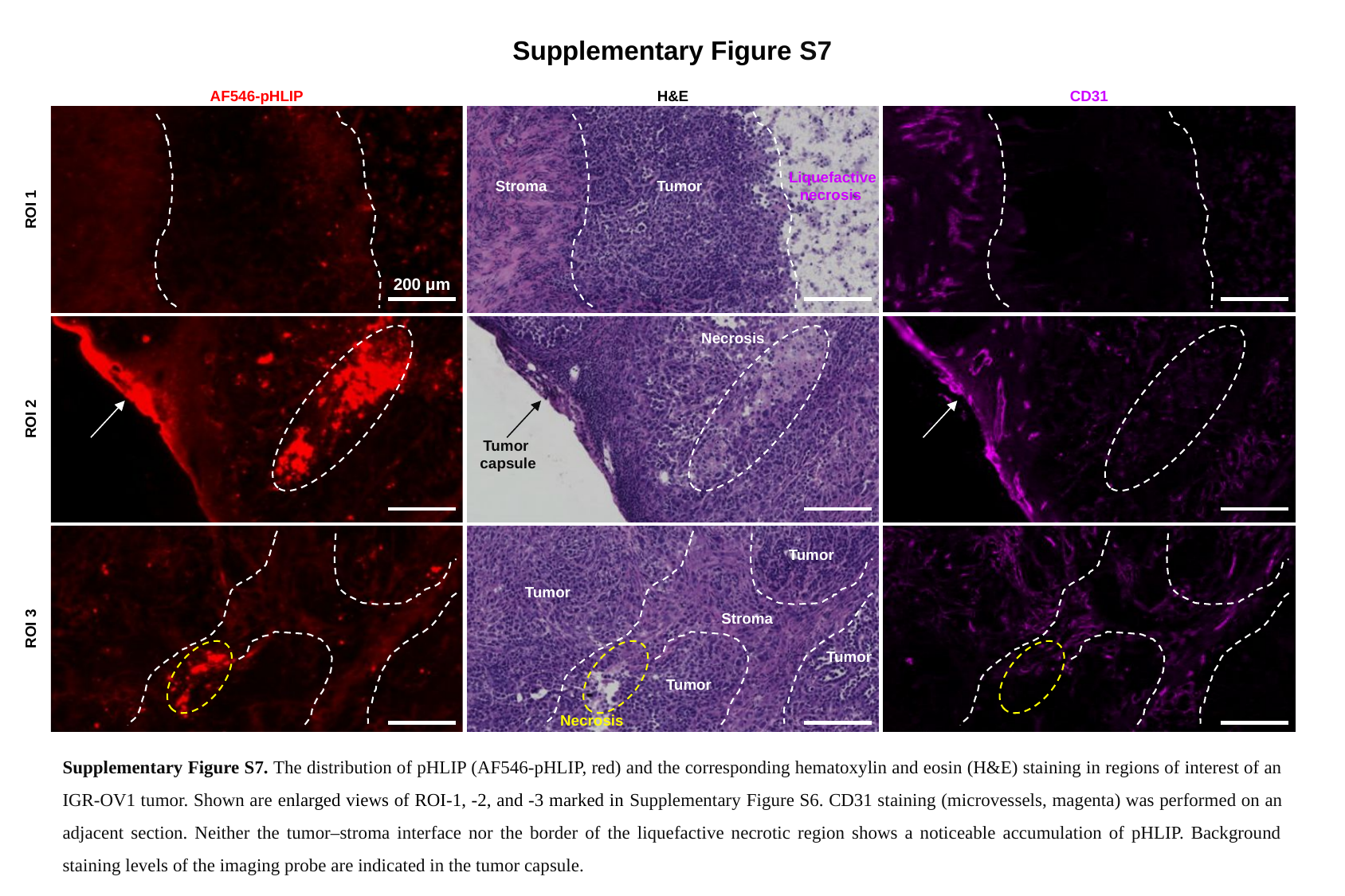

Supplementary Figure S7
AF546-pHLIP
H&E
CD31
Liquefactive
necrosis
Stroma
Tumor
ROI 1
200 μm
Necrosis
ROI 2
Tumor
capsule
Tumor
Tumor
Stroma
ROI 3
Tumor
Tumor
Necrosis
Supplementary Figure S7. The distribution of pHLIP (AF546-pHLIP, red) and the corresponding hematoxylin and eosin (H&E) staining in regions of interest of an IGR-OV1 tumor. Shown are enlarged views of ROI-1, -2, and -3 marked in Supplementary Figure S6. CD31 staining (microvessels, magenta) was performed on an adjacent section. Neither the tumor–stroma interface nor the border of the liquefactive necrotic region shows a noticeable accumulation of pHLIP. Background staining levels of the imaging probe are indicated in the tumor capsule.

## Slide 8
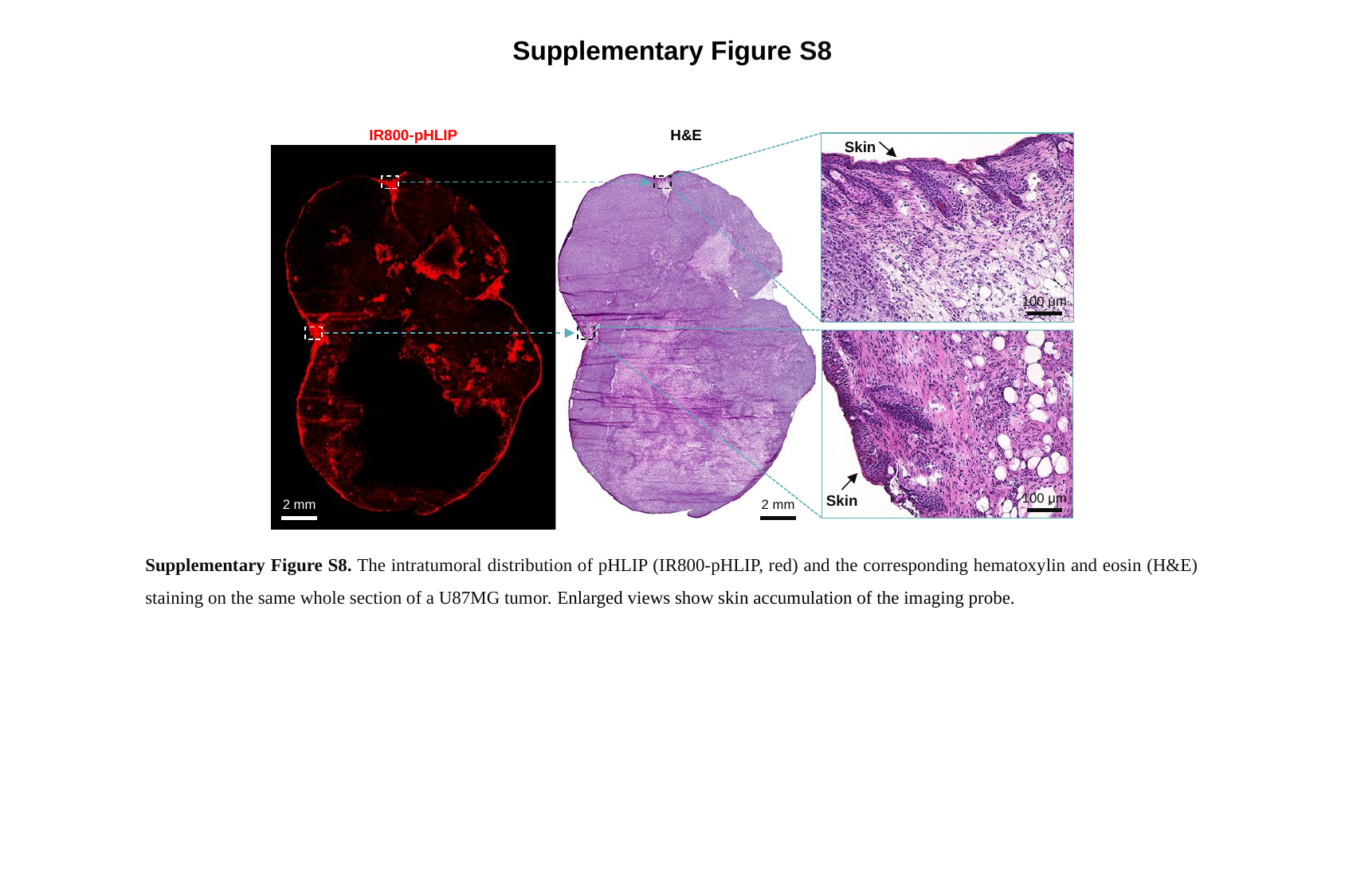

Supplementary Figure S8
IR800-pHLIP
H&E
Skin
100 μm
100 μm
Skin
2 mm
2 mm
Supplementary Figure S8. The intratumoral distribution of pHLIP (IR800-pHLIP, red) and the corresponding hematoxylin and eosin (H&E) staining on the same whole section of a U87MG tumor. Enlarged views show skin accumulation of the imaging probe.

## Slide 9
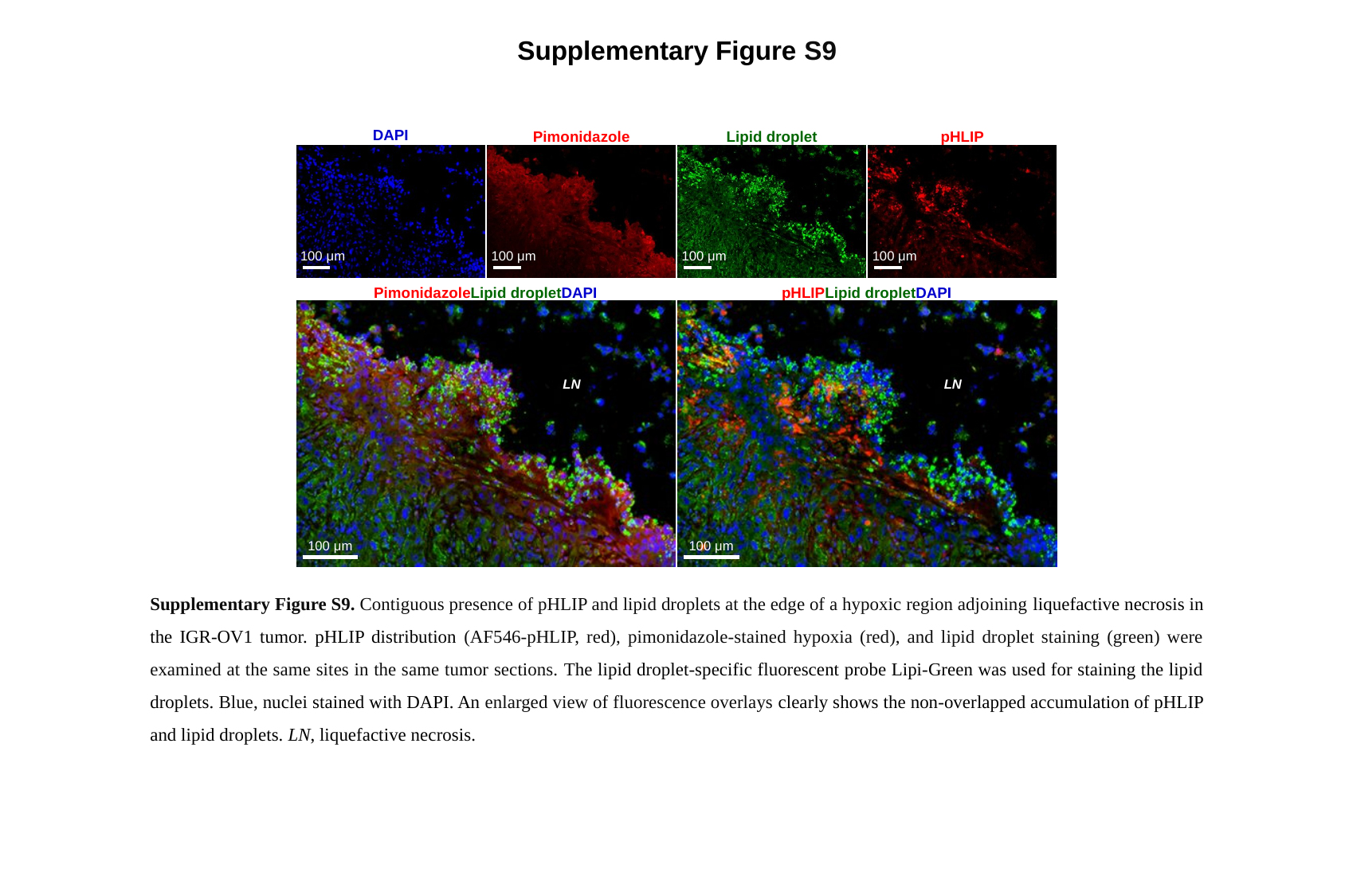

Supplementary Figure S9
DAPI
Pimonidazole
Lipid droplet
pHLIP
100 μm
100 μm
100 μm
100 μm
PimonidazoleLipid dropletDAPI
pHLIPLipid dropletDAPI
LN
100 μm
LN
100 μm
Supplementary Figure S9. Contiguous presence of pHLIP and lipid droplets at the edge of a hypoxic region adjoining liquefactive necrosis in the IGR-OV1 tumor. pHLIP distribution (AF546-pHLIP, red), pimonidazole-stained hypoxia (red), and lipid droplet staining (green) were examined at the same sites in the same tumor sections. The lipid droplet-specific fluorescent probe Lipi-Green was used for staining the lipid droplets. Blue, nuclei stained with DAPI. An enlarged view of fluorescence overlays clearly shows the non-overlapped accumulation of pHLIP and lipid droplets. LN, liquefactive necrosis.

## Slide 10
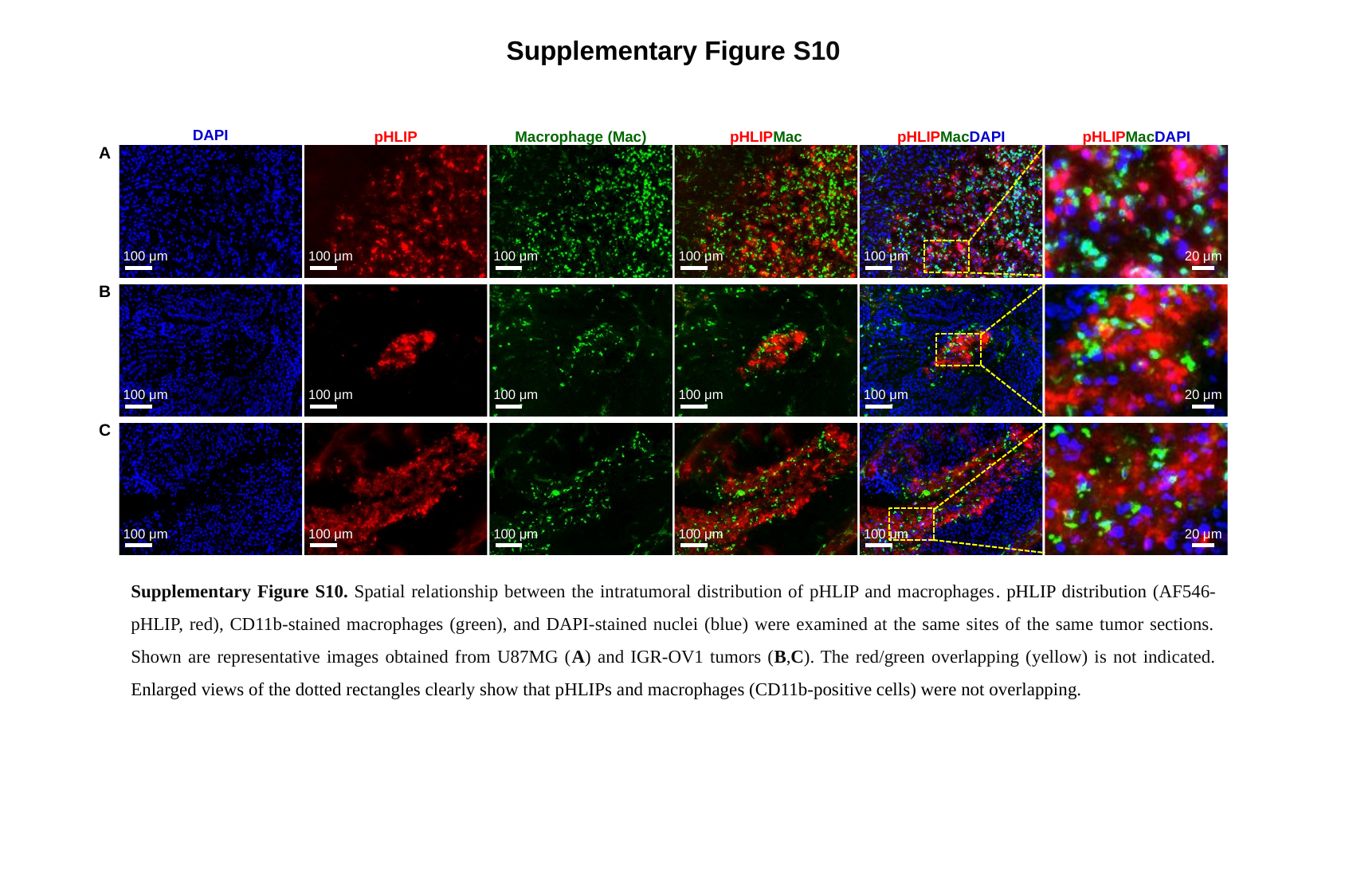

Supplementary Figure S10
DAPI
pHLIP
Macrophage (Mac)
pHLIPMac
pHLIPMacDAPI
pHLIPMacDAPI
A
100 μm
100 μm
100 μm
20 μm
100 μm
100 μm
B
20 μm
100 μm
100 μm
100 μm
100 μm
100 μm
C
20 μm
100 μm
100 μm
100 μm
100 μm
100 μm
Supplementary Figure S10. Spatial relationship between the intratumoral distribution of pHLIP and macrophages. pHLIP distribution (AF546-pHLIP, red), CD11b-stained macrophages (green), and DAPI-stained nuclei (blue) were examined at the same sites of the same tumor sections. Shown are representative images obtained from U87MG (A) and IGR-OV1 tumors (B,C). The red/green overlapping (yellow) is not indicated. Enlarged views of the dotted rectangles clearly show that pHLIPs and macrophages (CD11b-positive cells) were not overlapping.

## Slide 11
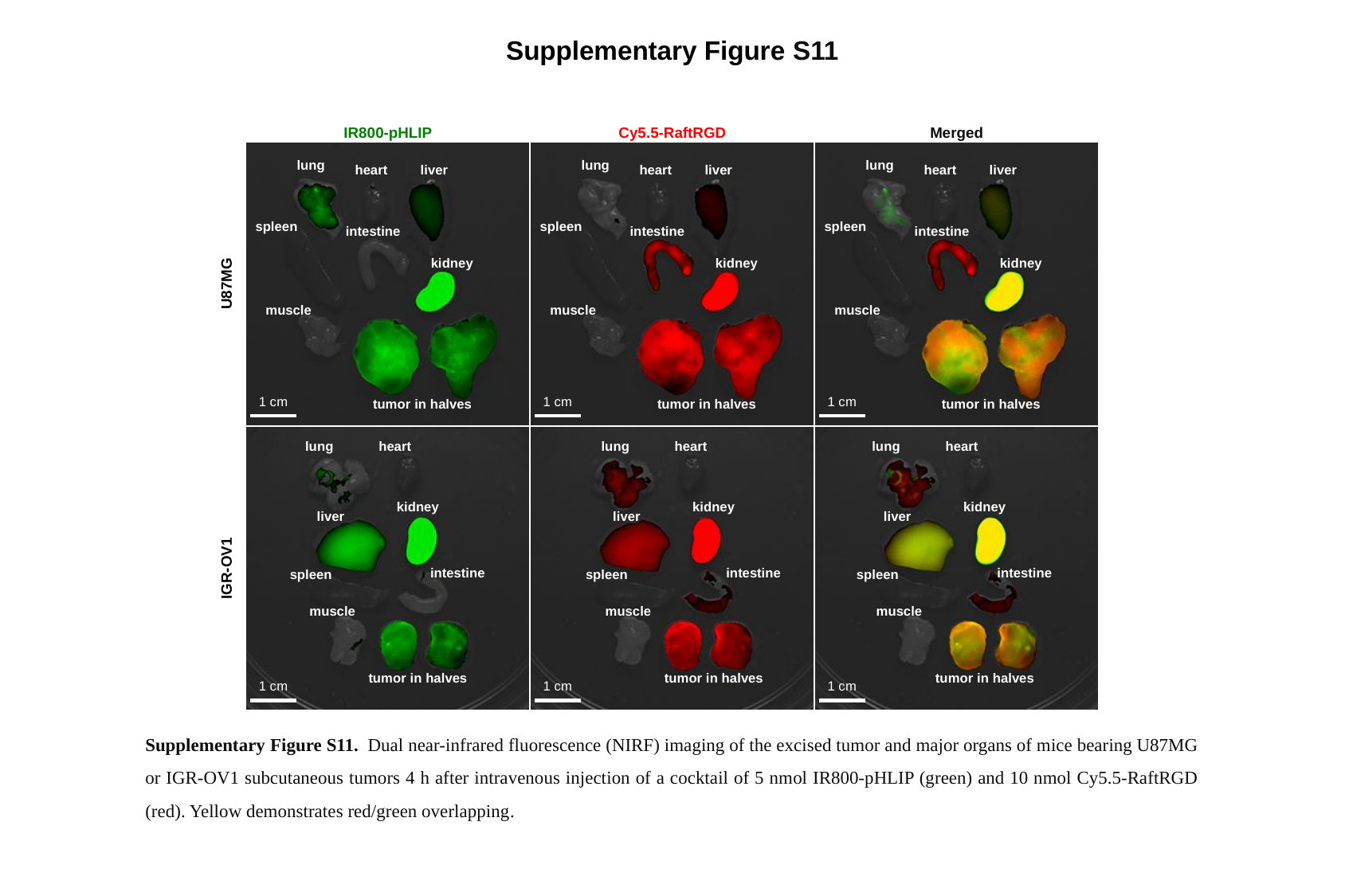

Supplementary Figure S11
IR800-pHLIP
lung
heart
liver
spleen
intestine
kidney
muscle
1 cm
tumor in halves
lung
heart
kidney
liver
intestine
spleen
muscle
tumor in halves
1 cm
Cy5.5-RaftRGD
lung
heart
liver
spleen
intestine
kidney
muscle
1 cm
tumor in halves
lung
heart
kidney
liver
intestine
spleen
muscle
tumor in halves
1 cm
Merged
lung
heart
liver
spleen
intestine
kidney
muscle
1 cm
tumor in halves
lung
heart
kidney
liver
intestine
spleen
muscle
tumor in halves
1 cm
U87MG
IGR-OV1
Supplementary Figure S11. Dual near-infrared fluorescence (NIRF) imaging of the excised tumor and major organs of mice bearing U87MG or IGR-OV1 subcutaneous tumors 4 h after intravenous injection of a cocktail of 5 nmol IR800-pHLIP (green) and 10 nmol Cy5.5-RaftRGD (red). Yellow demonstrates red/green overlapping.

## Slide 12
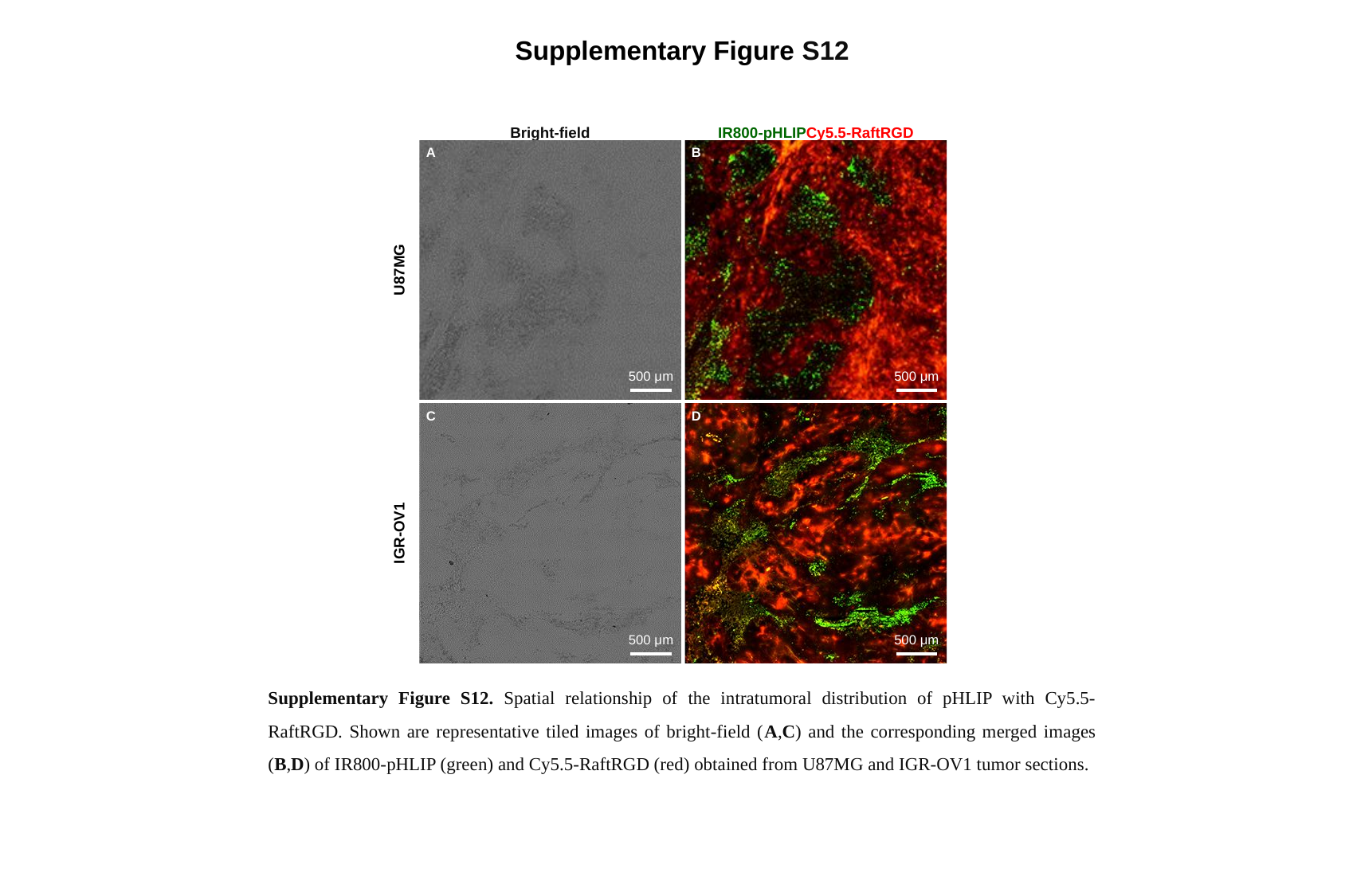

Supplementary Figure S12
Bright-field
IR800-pHLIPCy5.5-RaftRGD
A
500 μm
B
500 μm
U87MG
C
500 μm
D
500 μm
IGR-OV1
Supplementary Figure S12. Spatial relationship of the intratumoral distribution of pHLIP with Cy5.5-RaftRGD. Shown are representative tiled images of bright-field (A,C) and the corresponding merged images (B,D) of IR800-pHLIP (green) and Cy5.5-RaftRGD (red) obtained from U87MG and IGR-OV1 tumor sections.
